# Can DOT improve treatment-seeking behavior of TB patients?

**DOI:** 10.4103/0970-2113.63604

**Published:** 2010

**Authors:** Rajiv Paliwal

**Affiliations:** *Department of Chest Medicine, P.S. Medical College, Karamsad, Anand, Gujarat, India. E-mail: drrajivpaliwal@yahoo.co.in*

TB is an ancient disease. On March 24, 1882, Robert Koch announced the discovery of tubercle bacillus. He would have least expected that the world would be fighting hard to control TB, an easily curable disease, even after 125 years. In India, National TB programme (NTP) was formulated in 1962 and it functioned for almost three decades.[1] A review of NTP, in 1992, revealed managerial weakness, inadequate funding, over reliance on X-ray, use of non-standard treatment regimens and lack of systematic information on treatment outcome. Addressing these deficiencies, a revised National TB Control Program (RNTCP) incorporating the DOTS strategy was conceived. The RNTCP has been expanded in a phased manner rapidly from covering 18 million people in 1998 to the entire population of 1,114 million by March 2006.[2] TB is a communicable disease and requires a long treatment for at least six months. The longer duration of treatment poses a threat of poor treatment adherence and defaults, which is detrimental to the success of the TB control program. A number of community-based studies in different parts of India, have shown significantly high defaulter rate[3] even under DOTs strategy. Default is a Natural Phenomenon. The normal sensible person is the one who defaults. It is the abnormal, perhaps obsessive individual who continues to take medication for months after he/she feels Better.'[4] 'Default' is a human behavior. It reflects a poor treatment seeking behavior of TB patients. Directly observed treatment (DOT) makes it compulsory for a patient to swallow his/her medicines under direct observation. DOT being a technical intervention is less likely to improve treatment-seeking behavior of patients, which could be better influenced by aggressive health education and sensitization. In fact a study[5] comparing unsupervised treatment along with intensive health education and DOT concluded that the effect of intensive health education on the outcome of treatment is similar to that of direct observation of treatment.

Beyond DOT there are a few remedies and strategies which could help us improvise treatment compliance. Health Education is very crucial as compliance and adherence depend on various factors[6] like frequency of medications, number of pills, access to treatment, adverse drug reactions, symptomatic relief or lack of relief, complex treatment guidelines, dissatisfaction with treatment provider, poor communication and interaction etc. More over, in India, a sizeable proportion (around 60%) of cases being detected and treated solely in the private sector go unrecorded.[7,8] So the cases detected under the program do not, obviously, represent 70% of the TB burden. In that situation, 'Could the excellent cure rates reported among the comparatively fewer cases under the program lead to control of tuberculosis in the area?' The question needs to be answered. Health education and TB awareness could improve TB cure rates, irrespective of the treatment being provided in either (public or private) of the sectors. Continuing motivation and health education can reduce defaults at all stages of the treatment.[9] Aggressive health education and motivation could help to reduce default on account of migration and achieve the desired outcome in RNTCP.[10] There is an urgent need to improve the patient's perception of treatment and strengthen the health systems' capabilities to reduce initial default.[11]

Television and radio can be used for public service announcements and chat shows.[12] Social Marketing Techniques and Mass media network has the power and resources to improve the performance of TB control program. Community participation, which is essential for the success and sustainability of any program, has not happened as desired.[13] Studies[14] have shown a highly significant association between intensive health education and adherence to treatment. It has become apparent that without sustained social mobilization by conducting regular and effective IEC activities, achieving targets of 70% case detection rate and 85% successful treatment of such cases is not possible.[15] Extensive health education directed towards modifying patients perception of the illness, removing fear of discrimination and addressing a problem of stigmatization is needed to creat awareness and remove myths about tuberculosis in the community.[16] There is an urgent need to accelerate the development of 'New TB drugs' that could shorten the duration of treatment and improve compliance. The longer duration of treatment poses a threat of poor treatment adherence and defaults, which is detrimental to the success of the TB control program.
